# Boards of Examiners

**Published:** 1897-07

**Authors:** 


### BOARDS OF EXAMINERS.

---

### EXAMINATION FOR CITY VETERINARIAN, PHILADELPHIA, JUNE, 1897.

The questions are arranged so that they can be answered in a few lines. Do not go into great detail, but confine your replies to explicit statements and principles. Not more than one page should be devoted to a single question with its subdivisions.

1. *a*. Enumerate the principal symptoms of glanders, and three points of difference between glanders and colt distemper. *b*. What is the best treatment for glanders?

2. *a*. What are the symptoms of influenza? *b*. What are the complications of influenza? *c*. How should an uncomplicated case of influenza be treated?

3. *a*. What are the causes of pneumonia? *b*. What are the usual causes of death in pneumonia? *c*. Write one prescription suitable for a horse with pneumonia.

4. *a*. How may heat-stroke be recognized? *b*. If a horse with heat-stroke is down in the street, what should be done for him?

5. *a*. What is colic? *b*. How does colic cause death? *c*. Describe a case of colic that you have treated successfully, and your treatment for it.

6. *a*. What are the symptoms of founder? *b*. What is the treatment for founder in its earliest stages?

7. *a*. How should a horse with a corn be shod? *b*. How should a horse with sprained flexor-tendons be shod?

8. *a*. What should be done for a broken knee in which the joint is not opened? *b*. How should lacerated wounds be treated? *c*. How should a calk-wound be treated?

9. *a*. State the average normal temperature, pulse-rate, and respiration of a horse at rest. *b*. What should an examination for soundness include?

10. *a*. What are the principal defects of conformation to be avoided in a saddle-horse for police-service? *b*. Describe the type and conformation of a horse best fitted for service in a fire-engine team.

### PENNSYLVANIA STATE BOARD OF VETERINARY MEDICAL EXAMINERS.

The annual meeting and examinations by the Board were held at City Hall, Philadelphia, on June 21st and 22d, at 10 A.M. each day. The entire Board were present to conduct the examinations. Of the seventeen candidates presenting themselves for examination, four of these had failed at prior examinations; five of this number were graduates of the Ontario Veterinary College, and twelve represented the Veterinary Department of the University of Pennsylvania. Of this number sixteen passed and one failed. The following graduates of the Veterinary Department of the University of Pennsylvania obtained the license of the Board—Drs. Raymond Barber, Henry Bower, James Beatty, Edward O'Conner, Louis A. Klein, Augustus N. Lushington, John M. Montague, Edward M. Ranck, William G. Shaw, William J. Storm, William H. Smith, Jr., and Lloyd Zaner. Of the Ontario Veterinary College, Drs. J. C. Kingan, Robert W. McKibben, J. O. Reed, and Roy E. Jackson. With few exceptions, excellent marks were obtained. Reports of numerous cases now being prosecuted were brought before the Board and the action of the officers indorsed. Arrests of others were ordered and vigorous measures instituted. The re-election of Dr. W. Horace Hoskins as President, and Dr. S. J. J. Harger as Secretary followed. The Prothonotary of Lawrence County, who had illegally registered a graduate in September, 1895, had the same stricken off by order of the Court at the request of the Board. The members of the Board were entertained by the President at his home. The next examination by the Board will be held in December, 1897.

### VIRGINIA STATE BOARD OF VETERINARY MEDICAL EXAMINERS.

The examinations were held at Richmond, Va., June 23 and 24, 1897. The examinations were conducted by the entire Board. Two candidates presented themselves for examination—namely, Dr. A.

N. Lushington, graduate of the Veterinary Department of the University of Pennsylvania, and Dr. William H. Boyln, of Washington, D. C., a graduate of Toronto Veterinary College and of the Veterinary Department of the Columbian University.

---

### MARYLAND BOARD.

On June 29th, at Baltimore, the following veterinarians presented themselves for examination: Drs. J. S. Buckley, Mount Washington, graduate of the American Veterinary College; B. A. Brown, Cockeysville, graduate of Veterinary Department of Columbian University; Joseph N. Myers, Baltimore, graduate of Veterinary Department of Columbian University.

